# MicroCT analysis of vascular morphometry: a comparison of right lung lobes in the SUGEN/hypoxic rat model of pulmonary arterial hypertension

**DOI:** 10.1177/2045893217709001

**Published:** 2017-05-12

**Authors:** Erin M. Faight, Kostas Verdelis, Lee Zourelias, Rong Chong, Raymond L. Benza, Kelly J. Shields

**Affiliations:** 1 Lupus Center of Excellence – Autoimmunity Institute, Department of Medicine, Allegheny Health Network, Pittsburgh, PA, USA; 2 Division of Endodontics at the Department of Restorative Dentistry and Comprehensive Care and the Department of Oral Biology, University of Pittsburgh, Pittsburgh, PA, USA; 3 Cardiovascular Institute, Department of Medicine, Allegheny Health Network, Pittsburgh, PA, USA

**Keywords:** lung vasculature, microCT, Sugen-hypoxic rat model of PAH

## Abstract

Pulmonary arterial hypertension (PAH) is a rare disease characterized by significant vascular remodeling within the lung. Clinical computed tomography (CT) scans are routinely used to aid in PAH diagnosis. Animal models, including the Sugen-hypoxic rat model (SU/hyp), of PAH closely mimic human PAH development. We have previously used micro-computed tomography (microCT) to find extensive right lung vascular remodeling in the SU/hyp. We hypothesized that the individual right lung lobes may not contribute equally to overall lung vascular remodeling. Sprague-Dawley rats were subjected to a subcutaneous injection of vascular endothelial growth factor receptor blocker (Sugen 5416) and subsequently exposed to chronic hypoxic conditions (10% O_2_) for three weeks. Following perfusion of the lung vasculature with an opaque resin (Microfil), the right lung lobes were microCT-imaged with a 10-µm voxel resolution and 3D morphometry analysis was performed separately on each lobe. As expected, we found a significantly lower ratio of vascular volume to total lobe volume in the SU/hyp compared with the control, but only in the distal lobes (inferior: 0.23 [0.21–0.30] versus 0.35 [0.27–0.43], *P* = 0.02; accessory: 0.27 [0.25–0.33] versus 0.37 [0.29–0.43], *P* = 0.06). Overall, we observed significantly fewer continuous blood vessels and reduced vascular density while having greater vascular lumen diameters in the distal lobes of both groups (*P* < 0.05). In addition, the vascular separation within the SU/hyp lobes and the vascular surface area to volume ratio were significantly greater in the SU/hyp lobes compared with controls (*P* < 0.03). Results for the examined parameters support the overall extensive vascular remodeling in the SU/hyp model and suggest this may be lobe-dependent.

Pulmonary arterial hypertension (PAH) is a rare disease characterized by significant vascular remodeling within the lung. Computed tomography (CT) is routinely used to aid in PAH diagnosis. To date, clinical CT measures have been used to show differences in lung vascular volumes and patterns in smokers,^1^ those with emphysema,^2^ and chronic obstructive pulmonary disease (COPD).^3^ More recently, clinical CT scans were evaluated to quantify morphological biomarkers for diagnostic, phenotyping, and prognostic purposes in patients suffering from chronic thromboembolic pulmonary hypertension (CTEPH). The lung vasculature was segmented and 3D reconstructions were compared with patients without lung disease.^4^ CTEPH patients had greater pruning of the distal vasculature, greater proximal artery dilation, and increased tortuosity correlating with standard pulmonary hemodynamics.

Monitoring 3D morphological changes in lung vasculature of PAH patients using clinical CT scans would be preferred to more invasive hemodynamic measures. Animal models of disease are invaluable in determining mechanisms of pathological development. Several animal models of PAH exist; however, the Sugen-hypoxic (SU/hyp) rat model develops human-like plexiform lesions, which do not revert back to normal upon removal from hypoxic conditions.^5^ We have previously found evidence of extensive right lung vascular remodeling in the late stage SU/hyp rat model using micro-computed tomography (microCT).^6^ As a follow-up of this work, we hypothesized that individual lung lobes (1 = superior, 2 = middle, 3 = inferior, or 4 = accessory) may not contribute equally to overall lung vasculature differences observed in the SU/hyp model of PAH.

## Methods

### Animals

All experimental procedures were approved by the Institutional Animal Care and Use Committee. Adult male Sprague-Dawley rats weighing 180–200 g were either subcutaneously injected with SUGEN 5416 (20 mg/kg) and subsequently exposed to hypoxic conditions (10% O_2_) for three weeks^5^ followed by normoxic conditions (21% O_2_) for 12 weeks (SU/hyp: n = 11) or remained as control rats (control: n = 11) housed for the same length of time, but with no injection or hypoxic exposure.

### Tissue samples

Tissue sample collection was detailed previously.^6^ Briefly, rats were administered a sub-lethal dose of ketamine, the abdominal cavity was opened, the diaphragm was incised to expose the pleural cavity, and the ribs were cut away to access the heart. An incision was made in the right ventricle and polyethylene tubing (Tygon, 0.07 inch outside diameter) was inserted into the pulmonary artery and ligated. The circulatory system was flushed with 10 mL of heparinized saline using a syringe pump (Harvard Apparatus, PHD 2000). Immediately after the left pulmonary artery was exposed and ligated, the right lung was perfused with 10 mL radio-opaque silicone rubber polymer (Microfil, MW-122, yellow, Flow-Tech) for contrast enhancement during the microCT analysis, at a constant rate of 2 mL/min^–1^. The Microfil was allowed to polymerize for 24 h at 4℃. Right lungs were then fixed in 10% Neutral Buffered Formalin for a minimum of 48 h prior to microCT scanning.

### Micro-computed tomography (MicroCT)

Each of the four lobes of the right lungs were individually imaged using the Skyscan 1172 microCT (Bruker-Skyscan, Contich, Belgium) system with a 9.97 -µm voxel size and the following conditions: 55 kVp; 181 uA; 240 ms exposure; rotation step 0.250°; frame averaging 10; with a 360° rotation). The lung lobes were reconstructed from the raw files using NRECON (Skyscan Bruker) with a 40% beam hardening correction value and cone-beam reconstruction mode.

The imaged lobes were processed and analyzed separately (lobes proximal to distal: superior = lobe 1; middle = lobe 2; inferior = lobe 3; and accessory = lobe 4). Using CTAn software (Bruker-Skyscan), a region of interest (ROI) tracing the contour of each lung lobe, while excluding a uniform 750-µm-wide strip from the edge along the whole lobe height to eliminate edge defects, was generated by interpolation of similar user-defined ROIs on each lobe slice. Individual lobe regions of interest were then processed as separate datasets. The threshold was set at a grayscale value that was 25% lower than peak corresponding to the perfused pulmonary artery density distribution in a histogram of grayscale values within the ROI.

For the post-scan analysis of the imaged lung volumes, the following direct 3D measurements were performed: ratio of vascular volume to total lobe tissue volume; connectivity density; vascular number; vascular lumen diameter; vascular separation; and ratio of vascular surface area to vascular volume. These measurements are equivalent to the quantitative analysis of trabecular bone microarchitecture, which has been well documented, but applied to the lobe vasculature morphometry analysis within this body of work.^7,8^ The morphometry measurements were performed using the SCANCO 3D morphometry and densitometry software (SCANCO Medical, Brüttisellen Switzerland), operated in an OpenVMS environment. The same software was also used for generating 3D renderings of the lung vasculature lumen diameter maps.

### Statistical analysis

Normality of distribution for all covariates was assessed using the Shapiro–Wilk test. Parametric covariates were expressed as mean and standard deviation while non-parametric covariates were expressed as median and interquartile range (25th–75th%). Between-treatment (SU/hyp versus control at each lobe) and within-treatment (SU/hyp or control: superior versus middle versus inferior versus accessory) groups were analyzed using a Dunn’s test, which incorporates an initial omnibus, non-parametric Kruskal–Wallis test controlling for treatment and lung lobe followed by a post hoc multiple comparisons of independent groups using a Benjamini–Hochberg adjusted *P* value. All quantitative microCT parameters were evaluated using (Stata/IC 12.1, StatCorp, LP, College Station, TX, USA) with two-tailed *P* < 0.05.

## Results

**Fig. 1.**
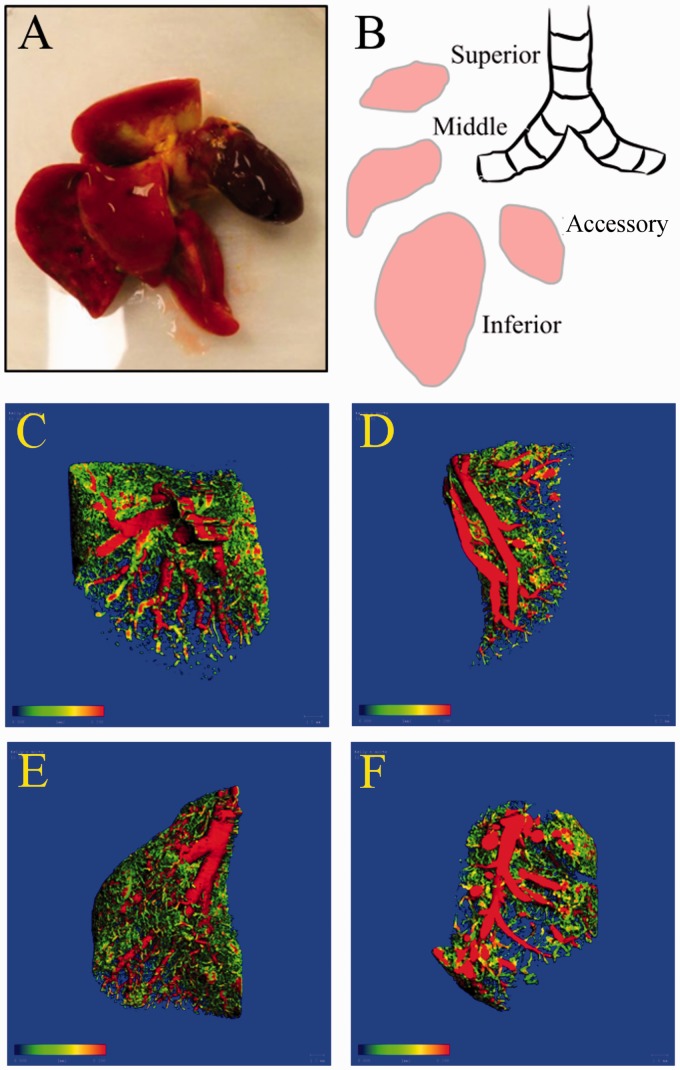
The right lung of the rat: (a) the right lung and heart with Microfil (yellow) is placed into 10% neutral buffered formalin before microCT scanning; (b) schematic of right lung lobes. Representative 3D images of SU/hyp right lung lobes: (c) superior = lobe 1, (d) middle = lobe 2, (e) inferior = lobe 3, (f) accessory = lobe 4 (scale bar range: 0.000 (blue) to 0.200 (red) mm).

We analyzed each distinct right lung lobe (superior = lobe 1, middle = lobe 2, inferior = lobe 3, accessory = lobe 4; Fig. 1a and b) separately using 3D thickness maps (Fig. 1c and d) and six different microCT parameters including: (1) the ratio of vascular volume to tissue volume (mm^3^/mm^3^); (2) connectivity density (a higher number signifies more vascular branching and normalized by tissue volume, 1/mm^3^); (3) vascular number (the number of continuous blood vessels at any point per unit length, 1/mm); (4) vascular lumen diameter (mm); (5) vascular separation (distance between the centers of adjacent blood vessels, mm); and (6) the ratio of vascular surface area to total vascular volume, mm^2^/mm^3^) for both the right control and SU/hyp lungs (Table 1).

**Table 1.** Comparison of microCT 3D morphometry parameter values for each lobe from the right lungs of control and SU/hyp animals.

	Control				SU/Hyp			
	Superior Lobe 1	Middle Lobe 2	Inferior Lobe 3	Accessory Lobe 4	Superior Lobe 1	Middle Lobe 2	Inferior Lobe 3	Accessory Lobe 4
Vascular volume/ tissue volume* (mm^3^/mm^3^)	0.333 (0.27–0.40)	0.304 (0.28–0.42)	0.347 (0.27–0.43)	0.373 (0.29–0.43)	0.236 (0.23–0.34)	0.261 (0.21–0.32)	0.233 (0.21–0.30)	0.273 (0.25–0.33)
Connectivity density^†^ (1/mm^3^)	51.8 (25–60)	18.9 (16–29)	44.7 (29–54)	21.5 (14–24)	41.3 (23–60)	31.5 (17–38)	22.0 (15–42)	24.1 (16–33)
Vascular number^‡^ (1/mm)	3.32 (3.0–3.7)	2.39 (2.3–2.7)	2.71 (2.4–2.9)	2.68 (2.6–2.8)	3.11 (2.3–3.2)	2.75 (2.5–3.0)	2.27 (2.2–2.5)	2.55 (2.2–2.7)
Vascular lumen diameter^§^ (mm)	0.305 (0.27–0.40)	0.507 (0.49–0.59)	0.550 (0.46–0.64)	0.552 (0.52–0.59)	0.356 (0.30–0.38)	0.419 (0.35–0.51)	0.594 (0.55–0.62)	0.482 (0.44–0.55)
Vascular separation** (mm)	0.264 (0.25–0.29)	0.350 (0.21–0.42)	0.271 (0.26–0.37)	0.287 (0.23–0.34)	0.346 (0.29–0.44)	0.338 (0.28–0.41)	0.384 (0.35–0.41)	0.365 (0.33–0.42)
Vascular surface area/ vascular volume^††^ (1/mm)	14.9 (14–19)	12.3 (12–13)	13.5 (12–15)	11.6 (11–12)	15.4 (14–17)	14.5 (14–16)	14.7 (13–16)	13.5 (13–15)

Values are presented as median (25–75%).

* Relative volume of vasculature in the lung lobe.

† Branching of vasculature, higher number signifies more vascular branching normalized by lobe volume.

‡ Number of continuous blood vessels at any point.

§ Blood vessel lumen diameter.

** Distance between the centers of adjacent blood vessels.

†† Relative surface area of the blood vessels related to total vascular volume.

### The distal lobes influence the ratio of vascular volume to tissue volume

The ratio of lobe-specific vascular volume to lobe-specific lung tissue volume between groups revealed no differences between the control and SU/hyp lobes 1 and 2, but a significantly greater ratio for control lobe 3 and the same trend for lobe 4 when compared with the SU/hyp lobes (*P* = 0.02 and 0.06, respectively). These findings suggest that any difference in the total right lung ratio of vascular volume to total lung tissue volume between the treatment groups is governed by the distal lobes and supports our previous findings of a greater ratio of vascular volume to total lung tissue volume within the control lung.^6^

**Fig. 2.**
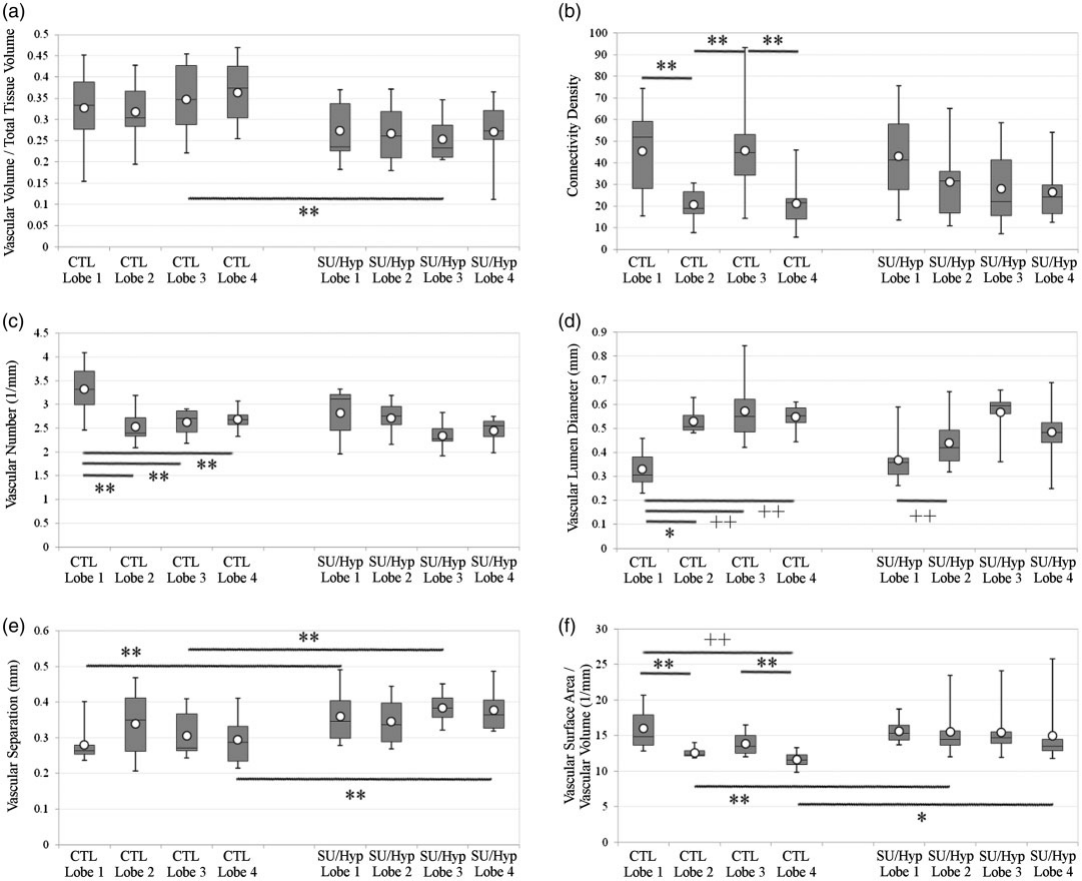
MicroCT parameters for each right lung lobe in both the control (CTL) and SU/hyp: (a) ratio of vascular volume to total lung tissue volume (mm^3^/mm^3^); (b) connectivity density (1/mm^3^); (c) vascular number (1/mm); (d) vascular lumen diameter (mm); (e) vascular separation (mm); (f) vascular surface area to vascular volume (mm^2^/mm^3^). Lobe 1 = superior lobe; lobe 2 = middle lobe; lobe 3 = inferior lobe; lobe 4 = accessory lobe. White dot = average. + *P* < 0.0001, + + *P* < 0.005, *P < 0.01, **P < 0.05.

No significant differences were found between lobes within each treatment group (Fig. 2a), which would indicate that the vascular volume of each lobe within the respective treatment group is being affected equally.

### The vascular connectivity density differs between lobes and is affected by SU/hyp treatment

The vasculature connectivity density highlights the extent of vascular branching normalized by the lobe volume within each lobe with higher numbers representing increased vascular branching. There were no significant differences between treatment groups for any of the lobes (Fig. 2b).

Within treatment group comparisons, overall the control group lobes were significantly different (*P* = 0.03). The post hoc analysis revealed a significantly greater connectivity density in lobes 1 and 3 when compared with lobes 2 and 4 (lobes: 1 versus 2 [*P* = 0.04], 2 versus 3 [*P* = 0.039], 3 versus 4 [*P* = 0.042]) with the same trend developing between lobe 1 and lobe 4 (*P* = 0.06). The connectivity density of the SU/hyp lobes were not significantly different (*P* > 0.4). The connectivity density findings are twofold: (1) differences in vascular density do exist between lung lobes with the superior (lobe 1) and inferior (lobe 3) lobes having a greater vascular network; and (2) the SU/hyp treatment attenuates the connectivity density of lung lobe 3 in particular (Table 1, Fig. 2b). These findings are remarkable considering that when we previously evaluated the vascular connectivity of the entire right lung^6^ we did not find any differences between control or SU/hyp groups, which underscores the possibility that there may be more nuanced differences within the separate lobes that we may miss when evaluating the lung as a whole.

### The vascular number is greatest in the superior lobe, regardless of treatment, but decreased overall in the SU/hyp group

The vascular number represents the density of continuous blood vessels per unit length of the lobe with higher numbers representing increased vascular continuity within the lung lobes. We found that control lobe 3 tended to have a greater vascular number when compared with SU/hyp lobe 3 in between treatment group comparisons (*P* = 0.07), while there were no significant differences between the other lung lobes 2, 3, or 4 (*P* > 0.1) (Fig. 2c).

Within treatment group comparisons, the control lung lobes overall had a significantly different vascular number (*P* = 0.014). In particular, lobe 1 continued to have a significantly greater number of continuous blood vessels when compared with the other lobes in post hoc analysis (lobes: 1 versus 2 *P* = 0.008, 1 versus 3 *P* = 0.023, 1 versus 4 *P* = 0.021). The same trend of the more proximal lobes having more continuous blood vessels when compared with the distal lobes continued in the SU/hyp group (*P* = 0.07) (Fig. 2c). These particular findings reveal two things: (1) the most proximal superior lobe has the greatest number of continuous vessels influencing the overall lung vascular number; and (2) while SU/hyp may not change the relationship between the superior lobes and the distal lobes, there is an overall decrease in vessel continuity due to the SU/hyp treatment (Table 1, Fig. 2c). These findings support our previous analysis, which revealed that the right control lung tended to have a greater vascular number,^6^ and this seems to be driven by the superior lobe.

### The vascular lumen diameter is greater in the distal lung lobes regardless of treatment

The vascular lumen diameter represents the blood vessel lumen diameter. We detected a trend for a greater vascular lumen diameter in control lobe 2 when compared with the SU/hyp lobe 2 (*P* = 0.06), but found no significant differences in vascular lumen diameter between the control and SU/hyp lung for individual lobes 1, 3, or 4 (all *P* > 0.1) (Fig. 2d).

However, we detected significantly greater vascular lumen diameters in the distal lung lobes for both control (lobes: 1 versus 2 *P* = 0.003, 1 versus 3 *P* = 0.002, and 1 versus 4 *P* = 0.001) and SU/hyp (lobes: 1 versus 3 *P* = 0.001), when compared with the more proximal lobes and, in particular, lobe 1. Additionally, this trend continued in the SU/hyp group when comparing lobe 1 with lobe 4 (*P* = 0.07) and lobe 2 with lobe 3 (*P* = 0.06) (Fig. 2d). These findings suggest that the vascular lumen diameter increases as we progress distally through the lung lobes regardless of treatment.

### The vascular separation is increased in the SU/hyp treatment group

The vascular separation represents the distance between the centers of adjacent blood vessels. Between treatment group comparisons reveal that vascular separation is greater in SU/hyp lung lobes 1 (*P* = 0.015), 3 (*P* = 0.021), and 4 (*P* = 0.027) when compared with the same control lobes (Table 1). No significant differences in vascular separation were detected between the control and SU/hyp lobe 2 (*P* = 0.9) (Fig. 2e).

Within treatment group comparisons revealed no significant differences in vascular separation for either control (*P* > 0.7) or SU/hyp (*P* > 0.6) lung lobes. These findings reveal that the SU/hyp treatment increases vascular separation driven by lobes 1, 3, and 4 with the possibility of sparing the upper middle lobe (lobe 2) (Fig. 2e) and support our previous finding of increased vascular separation in the right SU/hyp lung when compared to control.^6^

### The ratio of vascular surface area to vascular volume is greater in SU/hyp indicative of larger vessel diameters in combination with a reduction in vascular density potentially related to vascular pruning

The ratio of vascular surface area to vascular volume represents the relative surface area of the blood vessels relative to total vascular volume with higher numbers indicative of either larger vessels having a greater surface area or a reduced overall vascular density or a combination of both. The between treatment group differences revealed a significantly greater vascular surface area to vascular volume in the SU/hyp lobes 2 (*P* = 0.02) and 4 (*P* = 0.009) when compared with the same control lobes. There were no differences between SU/hyp and control lobes 1 or 3 (Fig. 2f).

Within treatment group comparisons, the ratio of vascular surface area to vascular volume was greater in the proximal lobes for the control group (lobes: 1 versus 2 *P* = 0.01, 1 versus 4 *P* = 0.0003, 3 versus 4 *P* = 0.016). There were no significant differences in vascular surface area to vascular volume between the SU/hyp lung lobes (*P* > 0.4) (Fig. 2f). These findings are twofold: (1) differences in the ratio of vascular surface area to volume exists between lobes in the control, indicative of lobe-specific differences in vascular diameters and density; and (2) the SU/hyp treatment attenuates lobe-specific differences in vascular surface area to volume, but this ratio is greater when compared to control. Coupled with our other findings, this suggests that SU/hyp treatment affects vascular diameter and volume differences between lobes, but promotes the loss of smaller vessels potentially related to vascular pruning. Again, these results support our previous findings in which we found the right SU/hyp tended to have a higher ratio of vascular surface area to vascular volume when compared to the control lung.^6^

### Vascular volume to total lobe volume as a function of vascular lumen diameter

**Fig. 3.**
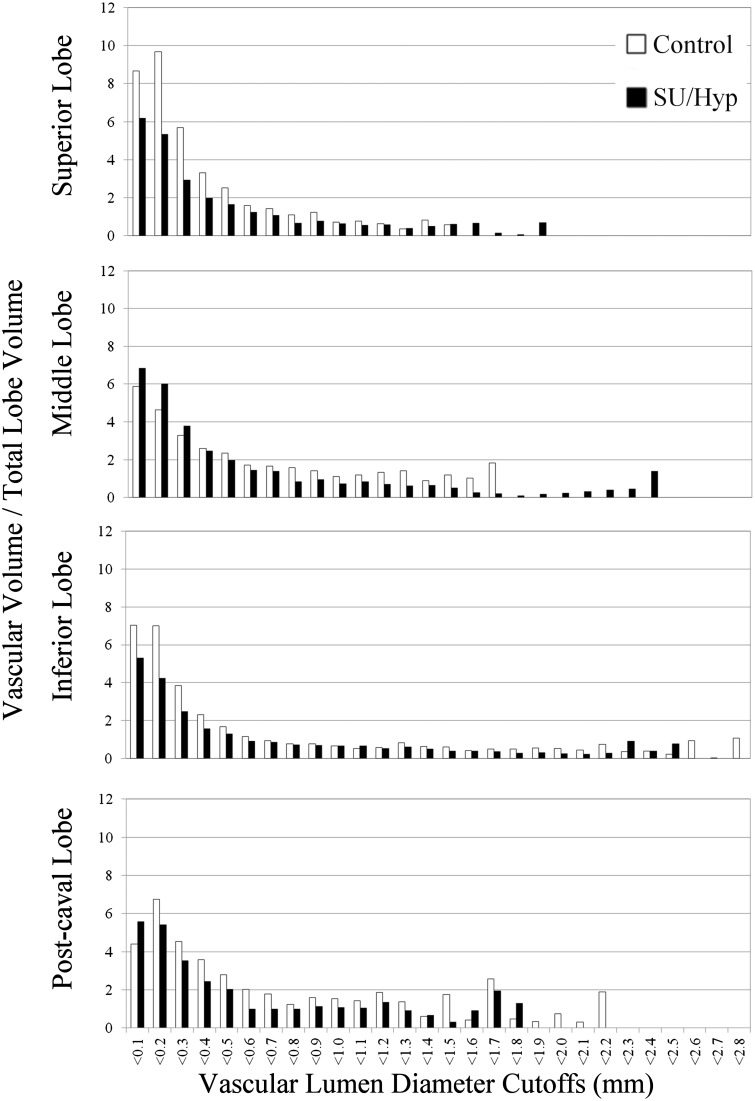
MicroCT ratio of vascular volume to total lobe volume as a function of vascular lumen diameter for each right lung lobe. Overall, the control lobes have greater vascular volumes at the smaller lumen diameters, while the SU/hyp lobes have greater vascular volumes at the larger lumen diameters. The middle and inferior lobes have a wider range of vascular lumen diameters for both the control and SU/hyp lobes.

Although we did not find a significant difference in vascular lumen diameter between the treatment groups, we did detect significant differences between the proximal and distal lobes within the treatment groups. We are able to graphically evaluate the relative vascular volume at specific vascular diameter ranges for each lobe thereby assessing a distribution of volumes to which each vessel diameter contributes (Fig. 3).

Overall, the control lobes tend to have greater vascular volumes at the smaller lumen diameters while the SU/hyp lobes have greater vascular volumes at the larger lumen diameters (Fig. 3). The middle and inferior lobes have a wider range of vascular lumen diameters for both treatment groups. The control superior lobe and the SU/hyp middle lobe have the highest frequency of smaller vascular lumen diameters (Fig. 3).

Our previous work also showed that average right lung lumen diameters were not significantly different between treatment groups; however, when we assessed the relative frequency of vascular lumen diameters in the same groups we were able to see a shift to larger diameters within the SU/hyp group likely reflective of vessel pruning and a higher prevalence of small diameter vasculature within the control group.^6^

## Discussion

MicroCT has traditionally been involved in the analysis of bones and teeth;^8,9^ however, its application to soft tissue, including lung vasculature,^6,10–12^ has been increasingly expanding. Arterial branching and total lung vasculature have been evaluated using microCT;^11,12^ however, this is the first to evaluate the contribution of the individual lung lobes to the overall vascular remodeling in the right lung of the SU/hyp rat model of PAH. Our findings indicate that not only does the SU/hyp treatment affect individual right lung lobes when compared to the control (control versus SU/hyp), but there also may exist vascular developmental differences between the lobes.

Pulmonary vascular development is a complicated process beginning in the embryonic stage of development through to the pseudoglandular stage.^13,14^ Several models have been proposed to describe vascular development in the lung including central angiogenesis with distal vasculogenesis from hematopoietic lakes^15^ or endothelial precursors^16^ along with distal angiogenesis.^14^ Endothelial cell (EC) lineage and heterogeneity is another area of intense study in the development of lung vasculature with some suggesting that the proximal regions of the lung are vascularized by ECs derived from the pulmonary trunk and artery while the distal regions are derived from the intersegmental arteries.^17,18^ Additionally, there are a multitude of molecular pathways, transcription factors and cellular expression have been implicated in the development of the lung vasculature such as vascular endothelial growth factor (VEGF),^19^ forkhead box factor family (Foxa1/2, Foxp1/2/4), and Wnt2 expressing cells.^20^ Our findings suggest that there are lobe-specific vascular distinctions in diameter, spatially, and density in the right lung of a rat, which may partially explain discrepancies in disease development and therapeutic responses between animal models of PAH and human PAH progression. These anatomic differences should be accounted for when evaluating rat models of pulmonary hypertension and the response to potential therapeutic interventions. Delineating the development of the individual lobes in the lungs may also aid in determining if certain lobes are more susceptible to disease manifestations and possibly better targets for treatment.

The progression of PAH has been noted by Rabinovitch et al. to develop distally and move proximally within the lungs.^21,22^ Others, including Bloodworth et al., have detailed that decreased distal pulmonary artery compliance may instigate a reduction in proximal pulmonary artery compliance with the development of PAH.^23^ As PAH progresses, patients experience vascular pruning of the lung which reduces the total vascular volume.^24,25^ Within the context of our microCT parameters, a result of vascular pruning includes not only a reduction in vascular volume, but can also be quantified by a reduction in vascular branching (connectivity density), decreased density of blood vessels (vascular number), and increased spacing between vessels (vascular separation).

X-ray CT methods were applied to quantitatively evaluate aspects of the pulmonary artery in rat lungs over a decade ago^26^ and Molthen et al. were one of the first to find a reduction in pulmonary vascular distensibility in Sprague-Dawley rats subjected to chronic hypoxic conditions using microCT.^27^ Adding to the current body of work we previously detected significantly greater ratios of vascular volume to total right lung tissue volume along with less vascular separation in control lungs when compared to SU/hyp lungs.^6^ Through this current analysis we were able to determine that the distal lobes of the SU/hyp rats 12 weeks post-hypoxic chamber drive these right lung differences. Scott et al. have now translated the power of microCT to human lung biopsy samples embedded with paraffin to compare with standard histology and assess 3D morphology.^28^

Quantifying aspects of the lumen diameter and the ratio of vascular surface area to vascular volume are also valuable parameters that can be obtained through 3D vascular thickness graphs of the lungs. We found the lobe-specific lumen diameter variation within both treatment groups with a shift to larger vascular diameters in the SU/hyp lobes when compared to the control lobes. These findings fit with known clinical measures such as dilated arteries in three or more lobes are 100% specific for the presence of pulmonary hypertension,^29^ and the clinical CT scans of CTEPH patients having greater pruning of distal vasculature while increased dilation of proximal arteries.^4^ Our study is not without limitations, which includes indirectly assessing changes in vascular pruning within the lung using various quantitative microCT vascular morphometry parameters. A rigorous microCT analysis must consider both the physical size of the sample and the length of scan duration to achieve a suitable resolution for analysis while maintaining a reasonably sized dataset. For this study, a reasonable resolution to consistently maintain was 20 um, which is the low threshold range for pulmonary vascular diameter in the rat.^30^ Inherent in any measurement is the possibility that the resolution of the system is not sufficiently high to capture the smallest vascular diameters and for this reason, the alterations to the lung vasculature at the periphery of the lung lobes and differences in the vascular diameters reflect the lung vasculature pruning process that is also observed in the human PAH condition, but may not prove the process. Additionally, no animal model fully replicates the intricacies of human pathology; however animal models are able to far better capture disease processes than the best computational models.^31^ Unlike other tried mouse and rat models of PAH, the SU/hyp rat model has replicated the human-like, PAH plexiform lesion development without regression of the disease process upon removal from hypoxic conditions.^5^ However, one must also consider the dependency of pulmonary circulation on gravity, which may play a role in the differences observed in vascular morphometry and disease pathology between rodent PAH models and upright humans.^32^ Considering our lobe-specific findings, it would also be beneficial to determine if there are vascular differences between the lungs and the individual lobes. Razavi et al. found a decreased overall lumen diameter and underdeveloped vascular branching after left pulmonary artery banding in a longitudinal evaluation of a rat model of pulmonary artery stenosis.^33^ Given the anatomical distinction in the number of lobes between the two lungs and our current lobe-specific findings future studies should also consider evaluating the effects on vascular remodeling between the two lungs and their lobes. This will eliminate the possibility of a within-animal sham, but also may prove to find a lobe-specific preferential pattern for lung disease development.

## Conclusions

We have shown overall differences in lung vascular remodeling^6^ and, within the context of this study, lobe dependent contributions to this overall vascular remodeling. Important questions arise from these findings: (1) is one lobe more susceptible to pathological changes to vascular morphology than the others? and (2) would more targeted therapy to a particular lobe be more appropriate? Further studies involving time points pre- and post-SU/hyp treatment are necessary to determine the development and rate of vascular remodeling using microCT.
